# Type IV Pili in Thermophilic Bacteria: Mechanisms and Ecological Implications

**DOI:** 10.3390/biom15040459

**Published:** 2025-03-21

**Authors:** Naoki A. Uemura, Daisuke Nakane

**Affiliations:** Department of Engineering Science, Graduate School of Informatics and Engineering, The University of Electro-Communications, Tokyo 182-8585, Japan; u2443002@gl.cc.uec.ac.jp

**Keywords:** type IV pili, rheotaxis, *Thermus thermophilus*, signal transduction, non-flagellated bacteria, optical microscopy, environmental microbiology

## Abstract

Type IV pili (T4P) machinery is critical for bacterial surface motility, protein secretion, and DNA uptake. This review highlights the ecological significance of T4P-dependent motility in *Thermus thermophilus*, a thermophilic bacterium isolated from hot springs. Unlike swimming motility, the T4P machinery enables bacteria to move over two-dimensional surfaces through repeated cycles of extension and retraction of pilus filaments. Notably, *T. thermophilus* exhibits upstream-directed migration under shear stress, known as rheotaxis, which appears to represent an adaptive strategy unique to thermophilic bacteria thriving in rapid water flows. Furthermore, T4P contributes to the capture of DNA and phages, indicating their multifunctionality in natural environments. Understanding the T4P dynamics provides insights into bacterial survival and evolution in extreme habitats.

## 1. Introduction

How fast do bacteria move? For *Salmonella*, a representative bacterium, the swimming speed is approximately 30 μm/s, corresponding to roughly 15-times its body length [1]. In general, swimming motility is a three-dimensional locomotion propelled by a flagellar motor that rotates helical filaments at its base [2,3]. However, bacteria have evolved alternative modes of locomotion that do not depend on flagella [4]. Many of these are categorized as surface motility, which is characterized by two-dimensional locomotion while remaining attached to a surface. The mechanisms underlying bacterial surface motility are remarkably diverse: some movements are driven by repeated cycles of extension and retraction of thin filaments known as type IV pili (T4P) [5], while others rely on helical tracks along the cell surface (leading to gliding motility) [6,7,8], and others depend on a specialized molecular machinery unique to *Mycoplasma* (leading to a process that is also called gliding motility) [9]. Some bacteria exhibit both swimming and surface motility, while others only exhibit surface motility. Notably, the instantaneous speed of this surface motility is approximately one-tenth or even less than that of swimming motility. Why have bacteria evolved such relatively slow surface motility? It has been hypothesized that in certain environmental contexts a slow migration style may be advantageous for survival. This review outlines the molecular mechanism of the T4P machinery, which is ubiquitous in both bacteria and Archaea and carries out a variety of functions, such as protein secretion, DNA uptake, and motility [10]. In particular, we focused on *Thermus thermophilus*, a non-flagellated biotechnological model bacterium isolated from a hot spring over 50 years ago [11], and on phylogenetically related thermophilic bacteria [12]. Based on our recent findings on long-distance migration in a rapidly flowing environment [13], we propose an ecological role for T4P-dependent surface motility in *T. thermophilus*.

## 2. Pili for Bacterial Surface Motility

Bacteria assemble various non-flagellar surface organelles termed pili [14]. These pili are thin filaments that either extend from the inner to the outer membranes or are localized in the outer membrane only. The pilus assembly machinery has diverse structures and functions involved in the biogenesis of different types of pili, such as T4P [5], chaperon-usher pili [15], conjugative type IV secretion pili [16], and the recently described type V pili [17]. Although there are many types of pili that are evolutionarily and structurally distinct, T4P are representative pili involved in force generation for bacterial surface motility.

## 3. Function Diversity of T4P

T4P play roles in protein secretion, attachment, surface motility, DNA uptake, and swimming motility [5,18,19]. These multifaceted properties are governed by a supramolecular complex embedded in the membrane, referred to as the T4P machinery [20,21,22]. Phylogenetic analyses of the T4P machineries from diverse prokaryotic genomes indicate that they can be classified into several groups [10]. T4aP and T4bP are responsible for surface motility, commonly referred to as twitching motility; Archaeal T4P is highly conserved in Archaea and associated with swimming motility; T2SS (type II secretion system) is conserved in diderm bacteria and specialized in protein secretion; and competence (Com) is responsible for DNA uptake in monoderm bacteria. Both T4aP and Archaeal T4P are involved in DNA uptake and motility. Comparative genomic analyses have suggested that T4aP can be found in *T. thermophilus* and related bacteria in the *Deinococcota* phylum (synon. *Deinococcus-Thermus* phylum) [10]. For simplicity, in this review the machinery classified as T4aP in *T. thermophilus* is referred to as T4P.

## 4. T4P Function in *T. thermophilus*

T4P in *T. thermophilus* has been reported to play a role in attachment, biofilm formation, DNA uptake, twitching motility, and phage infection [23,24,25]. As a highly thermophilic bacterium with an optimal growth temperature at 70 °C, research has predominantly focused on its extremophile biology [11,12,26], and its proteins have been widely utilized in numerous crystallographic structural analyses [27]. However, there have been few studies on the localization and dynamics of proteins at the single-cell level in thermophilic bacteria, especially in *T. thermophilus*. Remarkably, DNA uptake during transformation has been found to be more efficient in *T. thermophilus* than in other bacteria such as *Acinetobacter* [28]. Although direct visualization under high-temperature conditions presents significant challenges, *T. thermophilus* offers the potential for important and intriguing discoveries that are unlikely to be observed in common bacterial T4P systems.

## 5. T4P Components in *T. thermophilus*

**Figure 1:**
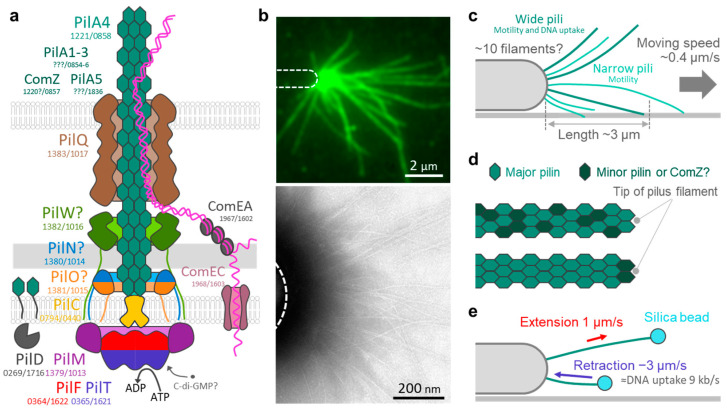
T4P in *T. thermophilus*. (**a**) Schematic representation of the T4P machinery, based on a diagram provided in a previous study [34]. Protein components are presented together with their gene IDs as identified in *T. thermophilus* strains HB8 (left) and HB27 (right). (**b**) T4P filaments were visualized by immunofluorescence microscopy of PilA (upper panel) and negative staining electron microscopy (lower panel). Cell outlines are indicated by dashed white lines. (**c**) Length, approximate number, and thickness of T4P filaments. (**d**) Minor and major pilin assembly into T4P filaments. (**e**) T4P dynamics. The T4P extension and retraction velocities were measured using microbeads as probes. Movements away from the cell pole were defined as positive values.

The T4P machinery in *T. thermophilus* is composed of several subcomplexes (Figure 1a). These include the outer membrane subcomplex PilQ; alignment subcomplex PilM, PilN, and PilO; the inner membrane platform protein PilC; and the cytoplasmic ATPases PilF (or PilB) and PilT [23,28,29,30]. In addition, proteins responsible for DNA uptake, such as ComEA and ComEC, are integrated into this machinery [28]. During T4P retraction, extracellular DNA is thought to be captured and pulled into the periplasmic space by ComEA; subsequently, single-stranded DNA is transported by ComEC to the cytoplasm [31,32,33]. Although similar routes have been proposed for cyanobacteria, *Vibrio*, and *Neisseria* [34,35,36], the detailed underlying mechanisms remain unclear. In *T. thermophilus*, two PilT homologs are present, with PilT1 functioning as the primary ATPase [23]. Owing to the characteristically wide periplasm in *T. thermophilus* [37], PilQ directly interacts with PilW [38], in addition to PilM, PilN, and PilO, to support its structure for proper localization of the T4P machinery [37,39,40].

PilA pilins are the major structural proteins of T4P filaments (Figure 1b). As do other pilins, PilA has an α-helix at its N-terminus [41,42]. PilA monomers polymerize to extend T4P filaments and depolymerize during retraction, diffusing over the surface of the inner membrane [43,44,45]. *T. thermophilus* has several paralogs of *pilA* in its genome [46]. Moreover, sequence diversity has been observed among *Thermus* strains [47]. Among these paralog proteins, PilA4 has been identified as the most functionally significant pilus subunit [48]. PilA1 and PilA3 are localized to the inner membrane, and PilA1 and PilA2 are implicated in DNA binding [29]. Importantly, PilA2 is thought to form a complex with ComZ [49], which is extruded through the outer membrane to directly interact with DNA [29]. Furthermore, PilA5 appears to be extensively involved in motility because its deletion does not affect DNA uptake [41]. However, these characteristics have been studied primarily in a standard strain, HB27. Whether similar features of the T4P machinery are conserved in other strains, such as HB8, has not yet been thoroughly investigated.

## 6. T4P Structure in *T. thermophilus*

Electron cryotomography of whole *T. thermophilus* HB27 cells revealed that pili are associated with large protein complexes crossing the periplasm (~70 nm) [37]. The structural size of the T4P machinery in *T. thermophilus* is approximately 20–30 nm longer than that of other bacteria [50,51]. The presence of at least 10 T4P machineries of the same type with a uniform size at one cell pole [37] suggests that one type of machinery is bifunctional for both motility and DNA uptake [52].

*T. thermophilus* forms two distinct types of T4P filaments, with thicknesses of approximately 7.0 nm and 4.5 nm each [41]. Wide pili are implicated in both motility and DNA uptake, whereas narrow pili—which are glycosylated—are exclusively associated with motility (Figure 1c) [41,53]. The ratio of the two pilus types varies with growth temperature, suggesting a temperature-dependent regulation of pilus expression [41].

Generally, minor pilins are not abundant, but are essential for the assembly of the pilus or for its specific functions [54] (Figure 1d); they can be further categorized into core and noncore minor pilins [55]. In *Pseudomonas* and *Myxococcus*, core minor pilins form complexes that stabilize the tip of the pilus and provide a template for the assembly of the major subunits [50,56,57]. In contrast, noncore minor pilins are involved in different biological functions [58], e.g., in *Neisseria* noncore minor pilins are incorporated throughout the pilus filament, presumably for antigenic variation to evade host immune responses [59]. However, the specific roles of minor pilins in mediating these functions in *T. thermophilus* remains unclear.

## 7. T4P Dynamics in *T. thermophilus*

We visualized T4P-dependent movements in single cells of *T. thermophilus* at their optimal growth temperature, 70 °C [13]. Under such conditions, bacterial movement is unidirectional, and cells travel more than 1 mm in an hour at a speed of 0.4 μm/s, which is relatively fast for T4P-dependent surface motility [60]. The T4P dynamics in *T. thermophilus* include velocities of 1 μm/s for extension and 3 μm/s for retraction (Figure 1e). These velocities were determined based on the ability of *T. thermophilus* to capture glass microbeads using T4P. This velocity is three-to-four-times faster than that of the mesophilic cyanobacteria *Synechocystis* but comparable to that of the thermophilic cyanobacteria *Thermosynechococcus*, both measured by the same method [61,62]. The speed of T4P-dependent movement is approximately 10% of the retraction velocity of a single T4P filament, indicating that the cell likely moves through the coordinated activity of multiple pilus filaments [63]. Given that a single pilus monomer contributes 1 nm to the filament length [41], approximately 1000 units should be added per second during polymerization and 3000 should be removed per second during depolymerization for moving.

Assuming that the retraction of a single T4P filament is coupled with the transport of a single-stranded DNA molecule of equivalent length, the velocity of DNA uptake was calculated to be 9 kbp/s per DNA translocation site. If a cell has four-to-five sites for DNA uptake, the velocity is approximately 40 kbp/s per cell, as previously reported by bulk biochemical measurements of DNA uptake in *T. thermophilus* [64]. Interestingly, natural competence is maintained in *T. thermophilus* even in the absence of PilT1 and PilT2 [23]. Moreover, PilT-independent T4P retraction has also been reported in other bacteria [65,66,67]. Therefore, further studies are required to elucidate the precise mechanisms underlying T4P dynamics and their association with DNA translocation.

## 8. T4P Polarity and Signal Transduction in *T. thermophilus*

In flagellated bacteria, directional movement is controlled by a two-component system that achieves a chemotactic response [68,69]. Homologous gene systems, Pil-Chp and Tax1, govern T4P-dependent surface motility by mediating chemotaxis in *Pseudomonas* and phototaxis in *Synechocystis*, respectively [70,71,72]. However, *T. thermophilus* lacks the corresponding genes in its genome, suggesting that these regulatory systems, which should facilitate its motility, are absent [46].

**Figure 2:**
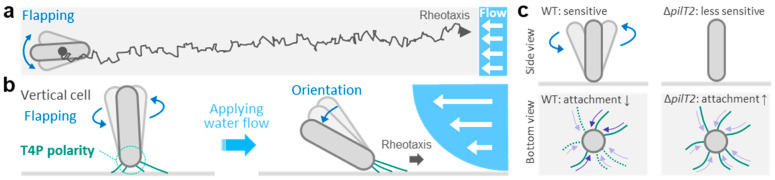
T4P-dependent rheotaxis in *T. thermophilus*. (**a**) Schematic representation of rheotaxis in *T. thermophilus*. (**b**) Schematic representation of T4P polarity and rheotaxis. In the absence of stimuli, cells are vertically attached to the surface at one of its poles and randomly flapping (**left**). In contrast, the cell axis orientates in parallel to the direction of water flow (**right**). (**c**) Putative function of dual motors needed for sensing the direction of water flow by facilitating the flapping motion of the vertical cell. The schemes represent the side and bottom views of a vertical cell during flapping.

The T4P machinery is typically localized at cell poles in rod-shaped bacteria, often with biased T4P activity at one pole [73,74,75]. An exception to this pattern is observed in *Thermosynechococcus*, which regulates asymmetric T4P distribution within a single cell pole [61]. Recent studies have reported that *T. thermophilus* exhibits positive rheotaxis, a directional movement against water flow [13] (Figure 2a). This T4P-dependent rheotaxis has also been reported in *Pseudomonas* and *Xylella* [60,76]. Therefore, the question should be asked of what the molecular mechanism involved in sensing the direction of water flow during rheotaxis is.

The initial step of the rheotactic response involves the vertical orientation of the cell body, indicating biased regulation of T4P activity at one cell pole (Figure 2b), as observed in *Pseudomonas* and *Myxococcus* [77,78]. In *T. thermophilus*, this vertical orientation is characteristic of nutrient-poor conditions. In *Myxococcus*, T4P polarity during surface motility involves MglA and MglB [79]. Genes encoding homologs to these proteins are present in *T. thermophilus* [46], and deletion mutants of these genes have no effect on pilus formation, but affect pilus localization at one cell pole [80], suggesting that these proteins play a role in T4P polarity. The second step is the weakening of the surface attachment, as observed in the flapping of the vertical cell (Figure 2b). The flapping motion is driven by the dual ATPases PilT1 and PilT2, which may produce an imbalance among multiple pilus retractions with distinct motor properties (Figure 2c). This hypothesis is consistent with the tug-of-war model proposed for T4P in spherical-shaped bacteria [63]. The resultant flapping motion allows the vertical cell to align its axis along the flow direction under shear flow. Subsequently, an asymmetric distribution of T4P against the direction of water flow should take place to achieve unidirectional cell migration.

## 9. T4P and Surface Sensing in *T. thermophilus*

T4P is important for surface sensing and the regulation of intracellular cyclic-di-GMP (c-di-GMP) levels [81,82]. PilB/PilF, an ATPase motor for T4P extension, features a characteristic sequence for the binding of this second messenger [83]. PilF in *T. thermophilus* binds to c-di-GMP. Notably, abolishing c-di-GMP reduces twitching motility and surface attachment, but not DNA uptake [84]. In cyanobacteria, T4P regulation is mediated by c-di-GMP over the short timescale of 1 min [61]. Assuming a similar timescale for T4P regulation in *T. thermophilus*, monitoring the intracellular concentration of c-di-GMP is of interest [85]. However, fluorescence measurements may not be applicable at high temperatures due to protein misfolding [86].

In *Pseudomonas*, surface sensing is thought to be coupled with temporal changes in the local PilA concentration in the inner membrane during T4P retraction [43,87]. In *T. thermophilus*, the average length and number of pili is 3 μm and 8 filaments, respectively (Figure 1b,c) [13], suggesting that at least 24,000 units of PilA are present per cell. Given the size of PilA on the inner membrane (~3 nm^2^), approximately 5% of the surface area of the cell body is occupied by the PilA monomer. Considering that the diffusion of the PilA monomer requires several seconds from pole to pole over the inner membrane surface and that T4P extension and retraction events occur roughly every 3 s [13], the local depletion of PilA at a cell pole could readily occur, as suggested for *Pseudomonas* [43]. Detailed biophysical measurements and physical modeling are required to fully understand surface sensing of T4P in *T. thermophilus*.

## 10. T4P Contributions to *T. thermophilus* Survival in Natural Habitats

**Figure 3:**
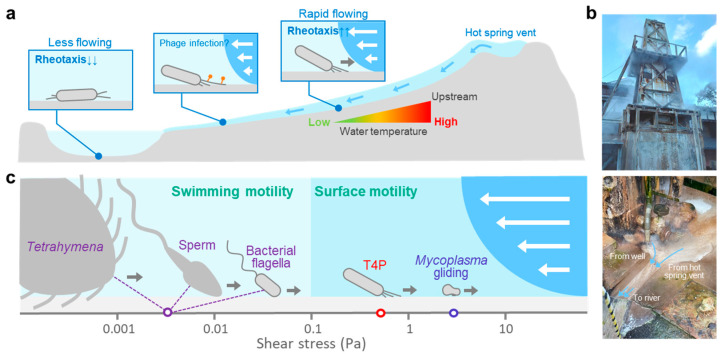
Rheotaxis of *T. thermophilus* in natural habitats. (**a**) Rheotaxis may have a role in flowing environments at hot spring vents. Low and rapid water flows may navigate cells to stay at (**left**) or move toward high temperature zones (**right**), respectively. The presence of T4P may facilitate infection by phages. (**b**) Symbolic building in the Mine Onsen hot spring (**top**). Hot spring water is drawn up from the source and used in baths and other facilities. The drawn hot spring water mixes with well water and some of it flows into the river (**bottom**). (**c**) Shear stress and rheotaxis in microorganisms, sperm cells, bacterial flagella, pili, and *Mycoplasma* gliding.

Notably, the role of T4P in the natural environment remains unclear. The rheotactic behavior of *T. thermophilus* HB8 enables it to travel long distances under rapid flow [13]. This ability may confer a significant advantage, allowing the bacterium to stay at or migrate closer to a hot spring vent (an environment with high temperatures), which may be favorable for its growth (Figure 3a). This seems consistent with the observation that rheotaxis is a characteristic of thermophilic bacteria, which is absent in the phylogenetically related mesophilic bacterium *Deinococcus* [88]. For example, the Mine Onsen hot spring, where *T. thermophilus* was originally isolated [11], emits hot spring water that flows into the river and mixes with cooler well water, reducing its temperature (Figure 3b). Such dynamic water currents likely drive the evolution of rheotaxis, enabling bacteria to adapt to flowing environments. In addition, variations in rheotactic activity among strains of *T. thermophilus* are noteworthy. HB27, which was isolated from the same hot spring as HB8, shows limited rheotactic activity [13], and AK1, isolated from the relatively stagnant water of the Arima hot spring [89], shows no rheotactic behavior [13]. This suggests that rheotaxis might represent an adaptation to rapidly flowing environments, whereas T4P in a slowly flowing environment may contribute to other cellular activities, such as aggregation.

Whether the rheotactic function of T4P extends to other thermophilic bacteria beyond *Thermus*, such as *Thermotoga* and *Aquifex*, remains an open question. These two thermophilic bacteria have genes related to T4P [90,91]; in *Thermotoga*, structures predicted to T4P machinery have been observed on the cell surface [92], yet T4P dynamics at the single-cell level have not been directly visualized. Furthermore, given that *Thermotoga* and *Aquifex* primarily rely on flagella-driven swimming motility [92], the ecological role of these T4P may differ from those in *Thermus*.

Additionally, T4P may contribute to the capture of small particles. The ability to capture glass particles indicates a potential role for T4P in biomineralization processes (Figure 1e). *T. thermophilus* TMY, isolated from silica scale [93], induces precipitation of supersaturated amorphous silica [94,95]. In contrast, phages, which are known to outnumber bacteria in the natural environment [96,97], often target T4P filaments during infection [98]. This mechanism has been well described in *Pseudomonas* [98], where the T4P retraction model was first proposed [99]. In *T. thermophilus*, infection by phages such as YS40 and TMA depends on the presence of T4P filaments on the host cell surface [24,100,101]. Considering that host specificity is different for both phages, the structural diversity of T4P filaments may have resulted from an evolutionary competition against each of these phages [24]. High-precision imaging of T4P dynamics during the capture of glass particles or phages can provide critical insights into the interplay of offensive and defensive mechanisms for bacterial survival strategies in natural environments (Figure 3a).

## 11. Conclusions and Perspectives

Rheotaxis, which is a response to mechanical stimuli, has been reported in various microorganisms [102]. The rheotactic response depends on the shear stress levels (Figure 3c), which are determined by factors such as body size and flow speed [103]. At shear stress conditions below 0.01 Pa, microorganisms (including eukaryotes and prokaryotes) can exhibit rheotaxis even while swimming [104,105,106,107], whereas at shear stress conditions higher than 0.1 Pa rheotaxis is characteristic of bacterial surface motility [60,76,108,109,110]. Rheotaxis may represent an adaptive response developed by microorganisms to locate and exploit ecological niches. Given that shear stress is influenced by the bacterial cell morphology, it is plausible that *T. thermophilus* and related thermophilic bacteria are sensitive to shear stress in the range of approximately 0.1–1.0 Pa (Figure 3c). Interestingly, the hot spring where *T. thermophilus* was isolated 50 years ago has a water flow with a shear stress of 0.1–1.0 Pa (Figure 3b), which is in good agreement with that in river ecosystems [111]. Several closely related species of *T. thermophilus* have been isolated from similar flowing environments, suggesting that water flow is a critical ecological factor with significant evolutionary implications for thermophilic bacteria. Visualizing these bacteria in flowing water environments offers insights into their adaptive strategies and ecological niches.

## Data Availability

All relevant data are included within this paper.
